# Comparative genomic prediction of resistance to Fusarium wilt (*Fusarium oxysporum* f. sp. *niveum* race 2) in watermelon: parametric and nonparametric approaches

**DOI:** 10.1007/s00122-024-04813-8

**Published:** 2025-01-24

**Authors:** Anju Biswas, Pat Wechter, Venkat Ganaparthi, Diego Jarquin, Shaker Kousik, Sandra Branham, Amnon Levi

**Affiliations:** 1 USDA, ARS, U.S. Vegetable Laboratory, 2700 Savannah Highway, Charleston, SC 29414 USA; 2 Coastal Research and Education Center, Clemson University, Charleston, SC 29414 USA; 3 University of Florida, Gainesville, FL 32611 USA

## Abstract

Complex traits influenced by multiple genes pose challenges for marker-assisted selection (MAS) in breeding. Genomic selection (GS) is a promising strategy for achieving higher genetic gains in quantitative traits by stacking favorable alleles into elite cultivars. Resistance to *Fusarium oxysporum* f. sp. *niveum* (*Fon*) race 2 in watermelon is a polygenic trait with moderate heritability. This study evaluated GS as an additional approach to quantitative trait loci (QTL) analysis/marker-assisted selection (MAS) for enhancing *Fon* race 2 resistance in elite watermelon cultivars. Objectives were to: (1) assess the accuracy of genomic prediction (GP) models for predicting *Fon* race 2 resistance in a F_2:3_ versus a recombinant inbred line (RIL) population, (2) rank and select families in each population based on genomic estimated breeding values (GEBVs) for developing testing populations, and (3) determined how many of the most superior families based on GEBV also have all QTL associated with *Fon* race 2 resistance. GBS-SNP data from genotyping-by-sequencing (GBS) for two populations were used, and parental line genome sequences were used as references. The GBLUP and random forest outperformed the other three parametric (GBLUP, Bayes B, Bayes LASSO) and three nonparametric AI (random forest, SVM linear, and SVM radial) models, with correlations of 0.48 and 0.68 in the F_2:3_ and RIL population, respectively. Selection intensities (SI) of 10%, 20%, and 30% showed that superior families with highest GEBV can also comprise all QTL associated with *Fon* race 2 resistance, highlighting GP efficacy in improving elite watermelon cultivars with polygenic traits of disease resistance.

## Introduction

Watermelon (*Citrullus lanatus*) is a major cucurbit crop, cultivated for its luscious fruit, high nutritional value, and economic importance (Massri and Labban 2014; Kousik et al. 2015). The domestication of this crop has focused on selective breeding for specific fruit traits. While these selective breeding efforts have resulted on modern watermelon cultivars with exceptional fruit quality, they inadvertently excluded alleles conferring resistance to biotic and abiotic stressors (Levi et al. 2001). Modern watermelon cultivars share a narrow genetic base due to many years of cultivation and selective breeding for superior fruit characteristics, rendering them less resistant to diseases and pests (Levi et al. 2001).

Fusarium wilt, caused by the fungus *Fusarium oxysporum* f. sp. *niveum* (*Fon*), is considered the major soil borne disease of watermelon. Besides *Fon* race 1, *Fon* race 2 has become most prevalent in all regions in the USA (Keinath et al. 2020). Over the past thirty years, it has spread worldwide wherever watermelon is grown (Bruton et al. 2008; Gonzalez-Torres et al. 1993). Fusarium wilt targets the vascular system of watermelon plants, leading to wilting, stunting, and ultimately plant death resulting in substantial yield losses. Chemical treatments are not sufficient in arresting the disease, and no edible watermelon varieties fully resistant to *Fon* race 2 have been developed (Ganaparthi et al. 2024). Although the development of varieties with durable resistance against *Fon* race 2 has been challenging for watermelon breeders, it is still be considered the most effective approach to combat the disease. Previous studies (Wechter et al. 2012; Levi et al. 2012) identified *Citrullus amarus* germplasm resources with resistance to *Fon* race 2 resistance. Subsequent studies using a F_2_:F_3_ and a recombinant inbred line (RIL) population derived from crosses between *Fon* race 2-resistant and susceptible *Citrullus amarus* accession, identified QTL associated with Fon race resistance using multiple QTL mapping (MQM) model (Branham et al. 2016, 2017, Branham et al.^2018^, Branham et al.2019; Ganaparthi et al. 2023). Ganaparthi et al. (2024) identified and validated one QTL on chromosomes 8 and two QTL on chromosome 9. While QTL associated with *Fon* race 2 resistance were identified and validated in *Citrullus amarus* germplasm (Branham et al. 2016, 2017, Branham et al.^2018^, Branham et al.2019; Ganaparthi et al. 2023), incorporating the resistance into watermelon cultivars (*Citrullus lanatus)* using marker-assisted selection (MAS) remains challenging due to its complex architecture (polygenic trait controlled by several gene loci with major and minor effects). Considering the *Fon* race 2 is complex trait and less predictive ability of QTL mapping, GS methods could be a viable option to develop disease-resistant trait especially *Fon* race 2 resistance in watermelon.

Incorporating *Fon* race 2 resistance from *Citrullus amarus* into watermelon cultivars (*Citrullus lanatus*) using marker-assisted selection (MAS) is challenging. This is since *Fon* race 2 resistance is a polygenic trait, controlled by gene loci with major and minor effects. While MAS mainly focuses on incorporating QTL with major effects, genomic selection (GS) (Meuwissen et al. 2001) could be useful in incorporating all *Fon* race 2 resistance QTL from the wild watermelon background into elite cultivars.

GS is a novel approach that uses machine learning algorithms handling complex genomic data, useful in harnessing genome-wide marker data to incorporate all or most gene loci associated with a polygenic trait (Meuwissen et al. 2001; Heffner et al. 2009; Bernardo 2014; He and Li 2020). Genomic estimated breeding values (GEBVs), computed from genomic prediction (GP) methods, are useful in predicting the genetic potential of each of the genotypes in a genetic population, and in streamlining the selection of most superior candidates in breeding programs (Jannink et al. 2010) to improve predictive ability or accuracy (PA) and genetic gain (Montesinos López et al. 2022; González-Recio et al. 2014). As with other crop species, GS holds the potential to improve quantitative traits of watermelon cultivars, including disease resistance, abiotic (drought, heat, and salt) stress tolerance and fruit quality. Here, the GP accuracy (the correlation between marker predicted value with true predicted genetic value) is calculated as the Pearson’s correlation between genomic estimated breeding value (GEBV) and the true (phenotypic) breeding value (Combs and Bernardo 2013; Isidro et al. 2015) to provide an estimate of selection accuracy (Merrick et al. 2022).

The selection intensity (SI) is a critical component in plant breeding. The probability of finding a superior genotype strongly depends on SI which indicates to what extent the selected group of genotypes is different from the overall population average. The selection intensity reflects whether the selected genotypes are from the top 10%, 5%, 1%, etc. of the population (i.e., percentile rank). The success of the breeding program strongly depends on the size of a population/number of individual genotypes that are being screened and selected for the desired trait (Marulanda et al. 2015). Effective GS programs have large training sets that facilitate high prediction accuracy and high SI, and consequently higher selection response/genetic gain (Gorjanc et al. 2017).

The research objectives of this study were to: (i) evaluate the effectiveness of various statistical models and frameworks for predicting and identifying the most resistant families in our two distinct *C. amarus* genetic populations (a F_2_:F_3_ and a RIL) segregating for resistance to *Fon* race 2 of watermelon, (ii) rank and select families using different SIs in each of these two populations based on their GEBVs, and (iii) verify if families with highest GEBVs also have all QTL associated with *Fon* race 2 resistance in these two populations. Families with the highest GEBVs and having all QTL associated with *Fon* race 2 resistance should be useful in further development of “testing populations” to incorporate the resistance into watermelon cultivars.

## Materials and methods

### Breeding populations

Two different intraspecific were examined in this study. Pop I (F_2:3_) was derived by crossing the *Fon* race 2-resistant USVL252^FR2^ with a susceptible PI 244019 (Branham et al. 2019b, a). Pop II (RIL; F_9_) was generated by crossing the *Fon* race 2-resistant USVL246^FR2^ with the highly susceptible inbred line USVL114 (derived by self-pollinating PI 542114) (Branham et al. 2017). All founder parents of these two genetic populations belong to the *C. amarus* (2n = 22) group collected in southern Africa and closely related and readily crossed with watermelon cultivars (*C. lanatus*) (Levi et al. 2012; Wu et al. 2019; 2023).

### Genotyping

The genomic DNA of the F_2_ parents of the F_3_ families (Pop I; Branham et al. 2017) and the F_9_ recombinant inbred lines (RILs) (Pop II; Branham et al. 2019b, a) undergone genotyping-by-sequencing (GBS) (Elshire et al. 2011) at Cornell University, Institute for Genomic Diversity. To ensure data quality, SNPs with more than 90% of missing values or a minor allele frequency (MAF) below 0.01 were filtered out using VCFtools version 0.1.15, as outlined by Danecek et al. (2011). Missing data were imputed using the window LD algorithm with the FSFHap plug-in, as described by Swarts et al. (2014) and available in TASSEL version 5.2.19 (Bradbury et al. 2007). Subsequently, consensus parental genotypes (A or B) were determined, removing any ambiguous or heterozygous loci, through the ABH plug-in in TASSEL. This process was based on the analysis of 10 sequencing samples from each parental line. The population genotypes (A, C, G, or T) were then transformed into consensus parental genotypes (A or B) (Branham et al. 2017; 2019b, a) and transformed into numerical genotypes for GP.

### Preparation of fungal inoculum

The preparation of *Fon* race 2 inoculum followed the optimized protocols outlined in Wechter et al. (2012) using the B05-30 isolate, generously provided by Professor Anthony Keinath of Clemson University. The race designation was confirmed by inoculating and assessing standard differentials for *Fon*, which included “Sugar Baby,” “All Sweet,” and PI 296341-FR (Branham et al. 2017; 2019b, a).

### Experimental design and evaluation of disease resistance

The F_2_:_3_ and RIL families (205 and 204 families in each, respectively) and their parents were evaluated in a greenhouse at the U.S. Vegetable Laboratory, Charleston, SC (75 °C day/60 °C night) The experimental design was a randomized complete block design (RCBD) with two replicates of 10 plants each, and two independent disease rating tests for both populations (i.e., a total of 40 F_3_ or RIL plants per family were tested). Plants were inoculated by seeding directly into a *Fon* race 2 inoculated soil (the soil was inoculated at the morning just prior to seeding) as described by Branham et al. (2017; 2019b, a). Phenotyping for resistance/susceptibility was conducted at 28 days post-inoculation (DPI) and plants were rated based on their disease severity. A plant that was completely symptomless was rated healthy “1”; while a plant that was slightly stunted/chlorotic was rated “2”; a plant with one or two wilted cotyledons was rated “3”; a completely wilted plant was rate “4”; while a dead plant was rated “5.”

### QTL analysis

We have used QTL analysis for two genetic populations we previously analyzed for identifying QTL associated with *Fon* race 2 resistance: Pop I (F_2_:F_3_; USVL 252FR2 x PI 482019) (Branham et al. 2019b, a) and Pop II (a F9 recombinant inbred line; RIL population; USVL 246FR2 x USVL 114) (Branham et al. 2017).

### Statistical model fitting

Since the non-additive variance (for dominance and epistasis) is minimal or zero for RIL population, and narrow sense heritability is more informative in GS, we calculated narrow sense heritability (h^2^) for both F_2_:_3_ and RIL population. Narrow sense heritability was computed for both populations using the ASReml-R package (Butler et al. 2009) in R (R Core Team, 2020). Linear mixed models were employed within the R software using the ASReml-R package (Butler et al. 2009) to derive eBLUEs for the fusarium wilt *Fon* race 2-resistant trait. The model employed for eBLUES calculation was:

$$y_{{{\text{ijk}}}} = \mu + t_{i} + r_{j} + f_{k} + e_{{{\text{ijk}}}}$$

where $y_{{{\text{ijk}}}}$ is the phenotypic value of the $k^{{{\text{th}}}}$ family, in the $i^{{{\text{th}}}}$ test and the $j^{{{\text{th}}}}$ replication. The term $\mu$ represents the population mean; $t$ is the random effect of the test (since two disease rating tests were performed for each population), with $t$ ~ *N*(0, $I\sigma_{t}^{2}$); $r$ is the random effect of replication, with $r$ ~ *N*(0, $I\sigma_{r}^{2}$); $f_{k}$ is the fixed effect of the $k^{{{\text{th}}}}$ family; and $e_{{{\text{ijk}}}}$ is the random effect of residual, with $e$ ~ N(0, $\sigma_{e}^{2}$). Estimated eBLUE’s for unaffected families having healthy plants for each test were later used to calculate the prediction ability (PA) of the GP models.

### Segregation observation

Histograms were constructed to examine the distribution pattern of each of the two distinct populations. The histograms were created using mean disease rating values, ranging from 1 to 5, where a rating of 1 represents completely healthy plants and a rating of 5 indicates dead or wilted plants.

### Genomic prediction models

The GP analyses were conducted considering six statistical models, and a tenfold cross-validation was implemented to evaluate PA. The prediction models were implemented in R using the BGLR package (Pérez and de los Campos 2014), along with caret (Kuhn 2020), glmnet (Friedman et al. 2010), random forest, e1071, and the AGHMatrix (Amadeu et al. 2016) that was implemented to compute the genomic relationship matrix G.

### Genotype main effects model

Consider that $y_{i}$ represents the phenotypic response corresponding to the *i*^th^ genotype derived from the eBLUEs previously computed. It can be explained as the sum of a common effect µ plus a random effect corresponding to the *i*th genotype $L_{i}$ (*i* = 1, 2,…, *n*) and error term $\varepsilon_{i}$ addressing the non-explained variability. The resulting linear model is as follows:

$$y_{i} = \mu + L_{i} + \varepsilon_{i}$$

where $L_{i} \sim N\left( {0, \sigma_{L}^{2} } \right)$, with $\sigma_{L}^{2}$ as the corresponding variance component, $\varepsilon_{i} \sim N\left( {0, \sigma_{\varepsilon }^{2} } \right)$, and $\sigma_{\varepsilon }^{2}$ as the variance component of the error term. One of the disadvantages of this model is that it does not allow the borrowing of information between genotypes because these are assumed to be independent and identically distributed (*IID*).

#### GBLUP model

The availability of marker SNPs allows the characterization of similarities between genotypes allowing the borrowing of information between calibration and testing sets. Consider that the genotype effect is replaced by a linear combination between *p* marker SNPs $x_{{{\text{ij}}}}$ (*j* = 1, 2,…, *p*) and their corresponding effects $b_{j}$ as follows $g_{i} = \mathop \sum \limits_{j = 1}^{p} x_{{{\text{ij}}}} b_{j}$ with $b_{j} \sim N\left( {0,\sigma_{b}^{2} } \right)$ and $\sigma_{b}^{2}$ as the associated variance component. Stacking all the genomic effects into a single vector ${\varvec{g}} = \left\{ {g_{i} } \right\}$ we have that ${\varvec{g}} = {\varvec{Xb}}$. Hence, by properties of the multivariate normal distribution, we have that the vector of genomics efcts follows a normal distribution centered on zero and with variance and covariance matrix proportional to ${\varvec{G}} = \frac{{XX^{\prime } }}{p}$ such that ${\varvec{g}}\sim N\left( {0, {\varvec{G}}\sigma_{g}^{2} } \right)$ and $\sigma_{g}^{2} = p\sigma_{b}^{2}$. Here, ${\varvec{G}}$ corresponds to the kinship matrix whose entries describe genomic similarities between pairs of individuals (VanRaden 2008). Collecting the previous results, we obtain the following linear predictor.

$$y_{i} = \mu + g_{i} + \varepsilon_{i}$$

This model was fitted using the R package BGLR version 1.0.5, and the AGHMatrix package was implemented to compute the ${\varvec{G}}$ matrix using the marker SNPs.

#### Bayes B and Bayes LASSO

The Bayes B (BB) and Bayesian LASSO (BL) models proposed by Meuwissen et al. (2001) were also implemented. These models differ in their prior distributions for marker effects, representing variations in genetic architecture. Their implementations were also conducted using the BGLR package. The study provides a comprehensive comparison of these Bayesian models, considering various factors such as marker variance, variable selection, and regularization parameter. Bayes B, a Bayesian method, improves prediction accuracy by considering the genetic architecture of complex traits influenced by a mixture of large and small effect markers.

#### Random forest

Random forest (RF) regression employs an ensemble of decision trees, each developed from a bootstrap sample of the training data and featuring randomly chosen subsets of predictor variables for node-splitting candidates. When predicting for a new observation *x*, the RF regression outcome is derived by averaging the outputs of the ensemble of *B* trees (Ogutu et al. 2011).

#### SVM linear and radial

Support vector machine (SVM) linear is the simplest form of the SVM kernel. It represents a linear decision between the classes in the feature space. In genomic selection where each feature corresponds to a genetic marker and the linear kernel tries to find a hyperplane that best separates the individuals with different phenotypic traits. SVM radial is a non-linear kernel that captures complex relationship between features. The radial basis function (RBF) kernel is more flexible and can handle non-linear relationships in genomic data. It can capture intricate genetic interactions that may not be apparent in a linear feature space. We used both statistical models in our experiment.

#### Model assessment

The GP models were evaluated using the Pearson’s correlation between the observed and predicted values based on tenfold cross-validation (CV). The CV scheme randomly assigned 90% of the families to the training set while the remaining 10% was used as validation set. Since the results derived from the tenfold CV varies depending on the random assignment of samples to the folds, this process was repeated 10 times to obtain consistent trends. The correlations obtained for each replicate and each fold were averaged and reported (average correlation based on 100 independent partitions). The predictive ability (PA) was obtained by calculating the Pearson correlation between predicted and adjusted phenotypes (eBLUE’s). In our study, predictive ability and predictive accuracy are the same as we genotyped the F_2_ generation (parent) and phenotyped the subsequent F_3_ generation (progenies), using them as a training set and validated the model performance accordingly.

#### Imposing selection intensities (SI) for capturing superior families and equating QTL analysis based on GEBV

Three different selection intensities (10% = 21 families, 20% = 41 families, and 30% = 61 families) were applied to determine how many superior families were captured based on GEBV. The GEBV results from GBLUP were compared with those families obtained using eBLUES to select the top-performing families. Subsequently we selected how many families contain major QTL associated with Fon race 2 resistance based on GEBV in both population: F_2_: F_3_ and the RIL.

## Results

### Phenotypic analysis

**Fig. 1:**
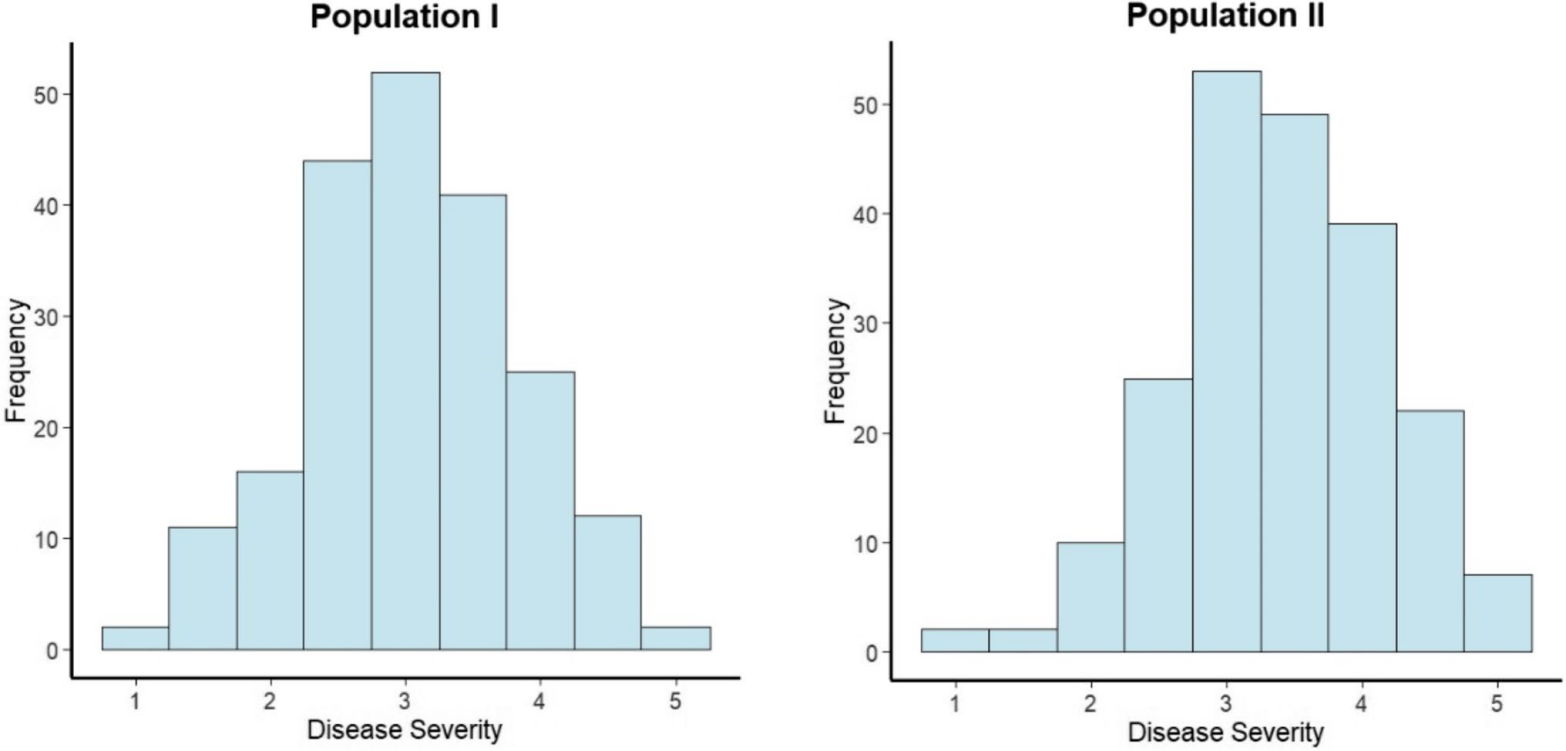
Histograms displaying the distribution of mean disease rating for each line from Population I (F_2_:F_3_; USVL252FR2 x PI 482019) and Population II (recombinant inbred lines; USVL 246FR2 x USVL 114) for disease severity (1–5; 1 = healthy, 2 = stunted/chlorotic, or 3 = one or two cotyledon is wilted, 4 = completely wilted, and 5 = dead) by *Fon* race 2 at 28 days after inoculation

In both populations, *Fon* race 2 resistance phenotypic distributions were approximately normal (Fig. 1.) Our top-performing genotypes based on different SI predominantly fell within the region marked “1 and 2” in the distribution plot (Fig. 1).

### Heritability

Moderate heritability of resistance to *Fon* race 2 exists in both populations examined in this study. Heritability indicates the proportion of the total phenotypic variability attributed to genetic factors in a population. It gives an estimation of the levels of predictive ability that can be reached predicting unobserved genotypes using GS when selecting for a targeted trait. For Pop I (F_2:3_), the heritability is 0.3, while for Pop II (RIL population) it was 0.42.

### Predictive ability

**Fig. 2:**
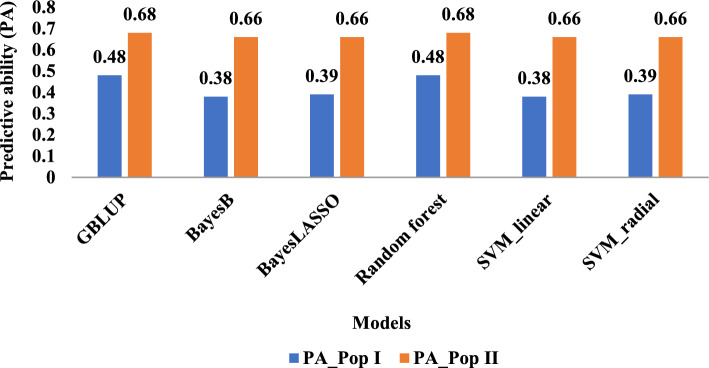
Predictive ability (PA) of six genomic prediction (GP) methods, three parametric (GBLUP, Bayes B, Bayes LASSO) and three nonparametric AI (random forest, SVM linear, and SVM radial) for two genetic/training populations: Pop I (F_2_:_3_) (PA_Pop I), and Pop II (RILs) (PA_PopII)

The predictive ability (PA) was consistent among the different models tested with these two populations (Fig. 2). For Pop I (F2:3), the values derived from the parametric and nonparametric AI methods ranged between 0.38 to 0.48, with GBLUP and random forest producing the highest correlation (0.48) between predicted and observed values (Fig. 2). For Pop II (RIL), the obtained PA values ranged from 0.66 to 0.68 across all prediction methods, while the GBLUP and random forest models produced a slightly higher PA value of 0.68 (Fig. 2).

### Comparison of coincidence of family selection based on GEBV

**Fig. 3:**
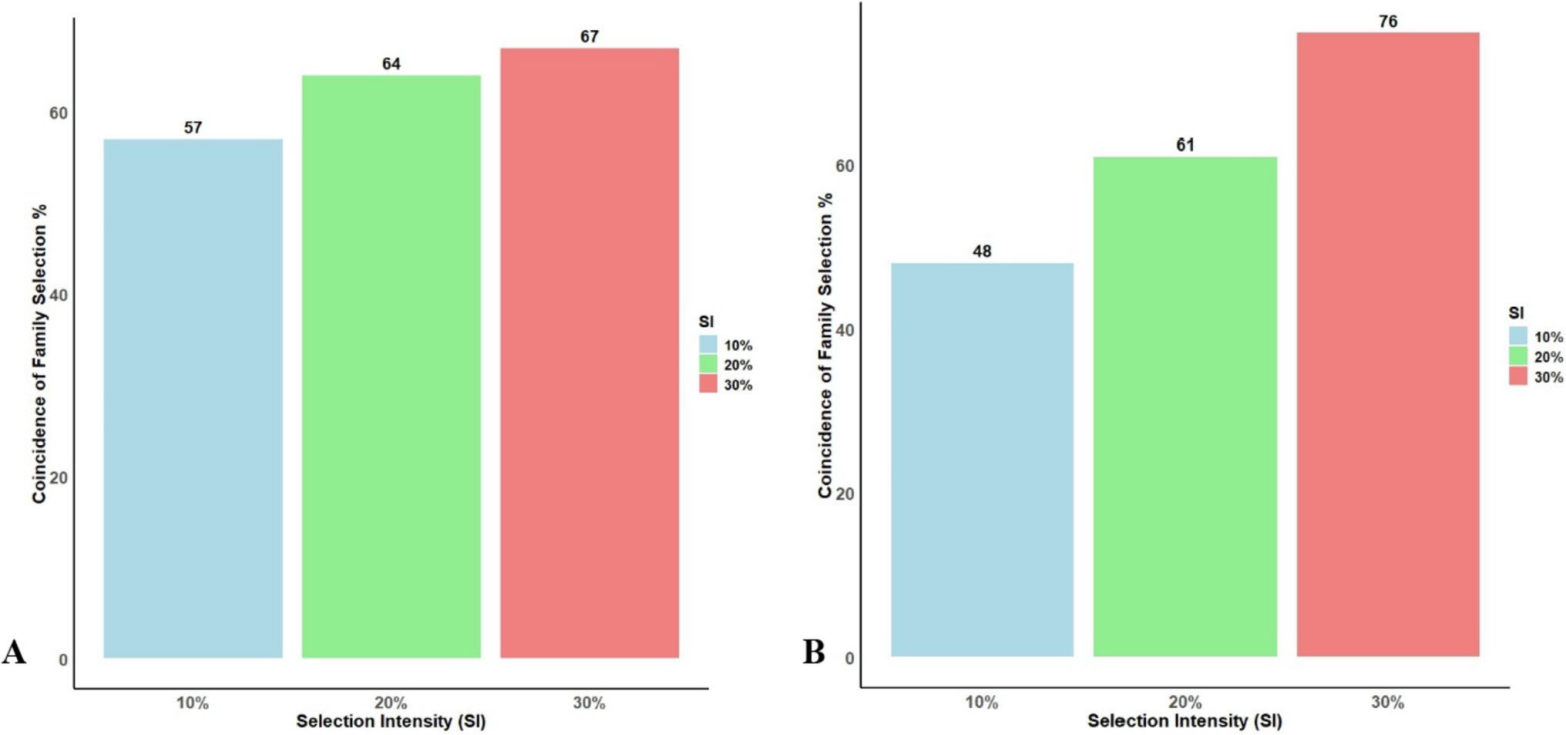
Percent coincidence of watermelon family selection of most superior families based on 10%, 20% and 30% selection intensity (SI) from genomic prediction vs phenotypic breeding value (eBLUEs) of A) pop I (F_2_:F_3_) and B) Pop II (RILs)

The coincidence of selection (referring to the degree to which the same individuals are selected using GS-GEBV versus phenotypic selection eBLUEs) varied significantly among the three selection intensities (Fig. 3). The 205 families of Pop I and 204 families of Pop II were ranked based on their eBLUEs and GEBV. The coincidence of family selection was relatively high for both populations at 30% SI (Fig. 3).

**Fig. 4:**
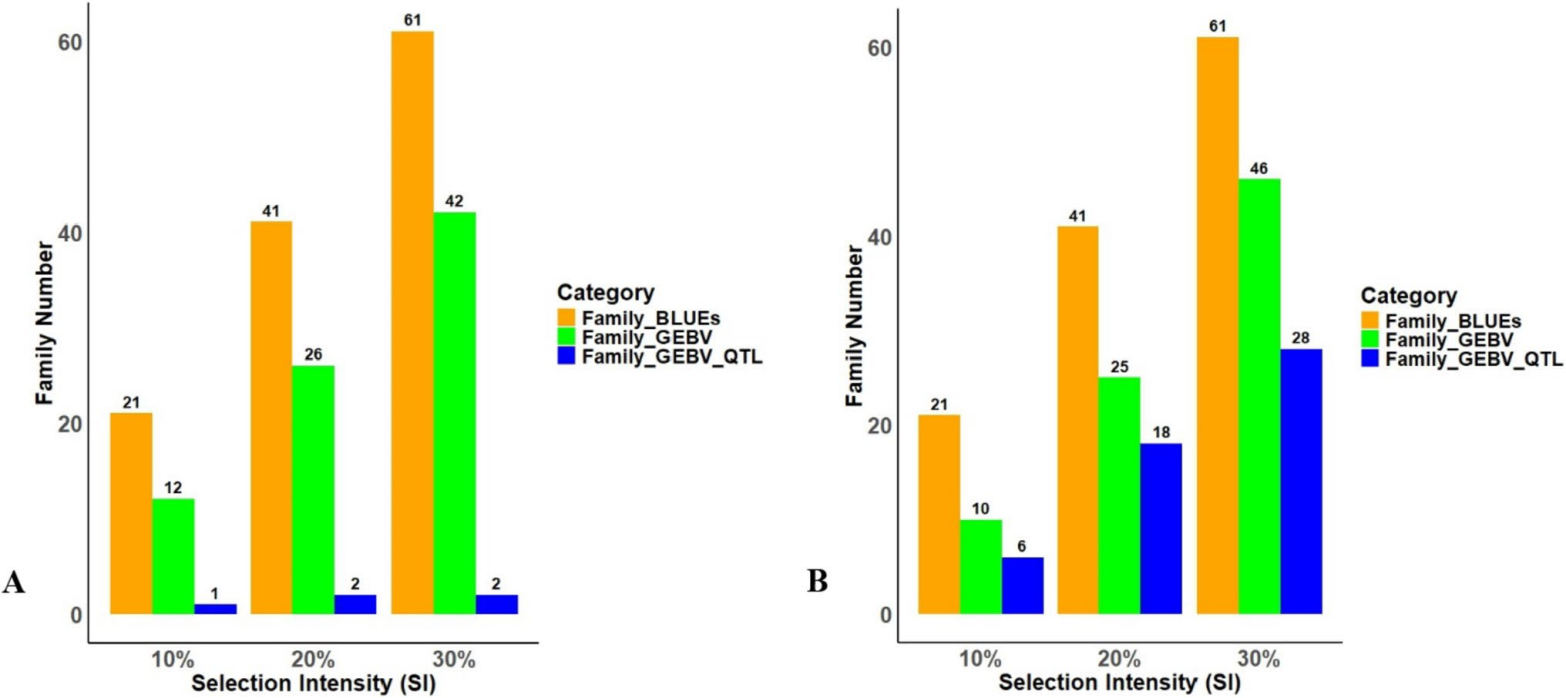
The number of superior families (genotypes) having the highest *Fon* race 2 resistance (orange color) selected out of 205 and 204 families in A) population Pop I (F_2_:_3)_ and B) Pop II (RIL) at different selection intensities (SI) (10%, 20%, and 30%). Of these, the number of families having the highest GEBVs (individuals selected based on coincidence of selection; using GS-GEBV versus phenotypic breeding value eBLUEs) (green color), and the number of selected families having highest GEBVs and comprising all quantitative trait loci (QTL) associated with *Fon* race 2 resistance (blue color)

In Pop I, GEBV captured 67% (42 families) of the superior families ranked by BLUEs when 30% SI was imposed, while 20% SI captured 64% (26 families), and 10% captured 57% (12 families) of these families (Fig. 3). On the other hand, in Pop II, GEBV captured 76% (46 families) of the superior families ranked by BLUEs when 30% SI was imposed, while 20% SI captured 61% (25 families), and 10% SI captured 48% (10 families) of these families (Fig. 4).

### Relating/equating QTL analysis and genomic selection

In Pop I (F_2_:_3_), the GS analysis captured alleles of the seven QTL (1 major on chromosome 1; and 6 minor QTL) associated with *Fon* race 2 resistance (identified in previous studies; Branham et al. 2016; 2017; 2019b, a) at the three different selection intensities (SI). In this population, three families captured all major and minor QTL. Using GEBV in Pop I (F_2_:_3_), 10 families were selected at 10% SI, and one of these 10 families comprised all QTL, while at both 20% and 30% SI, two families comprised all QTL (Fig. 4).

In Pop II (RIL), the number of families capturing resistance alleles of all three QTL (on chromosomes 8 and 9) varied across the three selection intensities (Fig. 4). At 10% SI, six out of 10 selected families comprised all three QTL. At 20% SI, 18 out of 25 families comprised all QTL, while at 30% SI, 28 out of 46 families comprised all three QTL. In this RIL population, a large proportion of families (69 out of 204) possess all three QTL associated with *Fon* race 2 resistance (Branham et al. 2016, 2018). Overall, the number of families selected varied across different SIs and between the two populations (Fig. 4).

## Discussion

GP is a valuable tool in plant breeding programs, facilitating efficient selection of superior genotypes for complex traits with low heritability (Xu et al. 2020). The success of GS in plant breeding largely depends on prediction accuracy, influenced by phenotypic, genetic, and mixed-model factors (Lopez et al. 2015). GP models can enhance selection accuracy and genetic gain while evaluating and selecting breeding populations (Heslot et al. 2012). The accuracy of GP is influenced by various factors, including the size of training population, trait complexity (heritability), marker density, statistical models, and genetic relationship between the training and testing population (Jia and Jannink 2012; Zhao et al. 2012; and Haile et al. 2018). However, limited research has compared the predictive abilities of different models in bi-parental populations of watermelon segregating for disease resistance. Our study focused on evaluating the predictive performance of various models in two population types: F_2:3_ and RIL populations.

### Prediction accuracy in the F_2_:_3_ versus RIL population

#### F_2_:_3_ population (Pop I)

In the F_2_:_3_ population, GBLUP outperformed other models, consistent with prior studies (Xavier 2019). Random forest also showed comparable performance, while the other methods (Bayes B, Bayes LASSO, SVM linear, and SVM radial) exhibited lower prediction accuracy. The effectiveness of GBLUP and RF likely stems from their ability to incorporate information across all genomic regions, a critical factor when predicting complex, disease-resistant traits in watermelon (Wang et al. 2015; Robertsen et al. 2019; Ghafouri-Kesbi et al. 2016). These models demonstrated high predictive accuracy, underscoring their utility for traits controlled by multiple loci.

#### RIL population (Pop II)

Pop II, consisting of RIL families, exhibited consistent prediction accuracy across various models, with an overall higher accuracy compared to Pop I. This outcome is attributed to the higher heritability and uniformity among individuals within the RIL families. Also, a high number of families in the RIL population (69 out of 204) possess all three QTL (two on chromosome 8 and one on chromosome 9) associated with *Fon* race 2 resistance compared with Pop I (F2:3) with only three families out of 205 families having all seven QTL (one major QTL on chromosome 1, and six minor QTL on chromosomes 2, 5, 6, 8 and 11). The RIL population’s stable genetic structure likely contributes to the high predictive power of GBLUP and random forest. These two models also had higher predictive power with the F_2:3_ population, further validating their robustness across different population structures.

#### Predictive ability of genomic prediction within populations with different methods

We observed that the RIL population consistently exhibited higher prediction accuracy compared with the F_2_:_3_ population across all models. The GP here reached up to 0.68 and 0.48 of accuracy for the RIL and F_2_:_3_ population, respectively. In our mapping studies with the RIL (Branham et al. 2016; 2017) and the F_2:3_ population (Branham et al. 2019b, a), the QTL in each of these two studies could together explain up to 43.2% and 42.7% of the genetic variation in *Fon* race 2 resistance. The high prediction accuracy in the RIL population is likely due to the high homozygosity among individuals in each of the RIL families and the high heritability of *Fon* race 2 resistance in this population. Heritability plays a significant role in prediction accuracy (Jia and Jannick, 2012; Desta and Ortiz 2014), while high heritability is positively correlated with higher PA (Desta and Ortiz 2014). Indeed, in our research here, the PA is higher in RIL population which has higher heritability value (h^2^ = 0.42) compared with the F_2:3_ (h^2^ = 0.3) in each statistical models we fitted for the GP.

A higher number of QTL with greater effect on a trait genetic variation should also improve PA. In our case, the RIL population has three QTL compared with the F2:3 population that have one major QTL and six minor QTL. The high number of families capturing all QTL (69 of 204 families) in the RIL compared with these in F2:3 population (3 of 205 families) could also contribute to the higher 0.68 versus 0.48 accuracy for the RIL and F2:3 population, respectively.

The computational efficiency in GS is critical for cross-validation and for model selection and assessment. Furthermore, the limitations imposed by scarce computational resources can present bottlenecks, particularly in the processing of extensive datasets. Overcoming these challenges requires the application of efficient statistical methods in GS processes (Ogutu et al. 2011). Recent advancements have diversified the statistical approaches employed to enhance predictive accuracy and model robustness. The combination of results obtained using parametric and nonparametric AI methods via assembling, holds the potential to improve GP in crop breeding programs (Mahood et al. 2020; Azevedo et al. 2024).

Adaptable to varying degrees of polygenicity, Bayes B is valuable for prioritizing markers with larger effects while accommodating numerous markers with smaller effects (Wang et al. 2015). The Bayesian Lasso stands out as a promising choice for genomic selection due to its simplicity, computational efficiency, and minimal requirement for prior information (Legarra et al. 2011). Additionally, the exponential distribution within the Lasso is considered to reasonably capture the nature of quantitative trait locus (QTL) effects (Goddard 2009). The Lasso, whether Bayesian or not, offers compelling advantages in the context of genomic selection. Bayes Lasso, a Bayesian adaptation of the Lasso method, performs variable selection and regularization concurrently. Suited for high-dimensional genomic data, it excels in traits influenced by a reduced number of markers with potentially varying effect sizes.

Support vector machine (SVM) with a linear kernel is effective when there is a clear linear separation between phenotypic categories, particularly suitable for traits with additive and linear relationships (Yang et al. 2018). SVM (support vector machine) is a standard nonparametric method employed for both classification and regression analysis in supervised learning. It adheres to the principle of structural risk minimization, considering both the fitting and complexity of training samples. Notably, SVM has found applications in GP in recent years (Ghafouri-Kesbi et al. 2017).

Genomic best linear unbiased prediction (GBLUP) is a widely used statistical method employed in animal and plant breeding to predict the genetic merit of individuals for specific traits (Heslot et al. 2012) and proved effective for predicting GEBV (Lorenzana and Bernardo 2009). It combines high-density genotypic information across the entire genome, offering more accurate predictions than traditional selection methods based on phenotypic and pedigree data (Jonas and de Koning 2013). This method uses straightforward statistical approach and efficient algorithms, making it accessible and computationally effective for large genomic datasets compared to other models. The RF is also a powerful machine learning ensemble method adapted for GP which is kinship based (Poland et al. 2012). Leveraging multiple decision trees, it excels in predicting complex traits, offering flexibility and success in diverse breeding scenarios which we observed in our study. Our results from different parametric and nonparametric GP methods indicate the high potential of GS in improving disease resistance in watermelon cultivars.

#### Selection intensity on QTL capture and superior family prediction

SI plays a crucial role in the effectiveness of GS by influencing the number of QTL captured and the identification of superior families. In Pop I, high SI of 10% identified 57% and 20% identified 64% of families of the 204 families in the population as superior, while only one or two families comprised all QTL. The lower SI of 30% captured 67% superior families and two families comprised all QTL. Previous study mentioned a single major QTL explained 18.9% of the variation while three minor QTL cumulatively explained 23.8% of variation for *Fon* race 2 resistance (Branham et al. 2019b, a). In the study, using GP methods we can predict up to 48% of genetic variation related to *Fon* race 2 resistance.

Conversely, in Pop II, a 30% SI captured 76% superior families, and 28 families comprised of all three QTL of 205 families, 20% SI captured 61% superior families and 18 families containing all three QTL and the 10% SI captured 48% families, and 6 families comprised of all three QTL. These findings suggest that higher SIs in certain populations can focus on major QTL while lower intensities may capture a broader spectrum of genetic variation. Notably, even with lower identified superior families, a 10% or 20% SI could still detect half, or more than half of the top families comprising all previously identified QTL in both populations, supporting the use of GS to efficiently screen large populations. The previous study for this population mentioned that the three major and minor QTL explained 28.2% of genetic variation for *Fon* race 2 resistance (Ganaparthi et al. 2024). In the study, using GP methods we can predict up to 68% of genetic variation. This higher genetic variation for both populations might be due to the presence of all minor QTL inclusion in GP methods that are not being captured by the MQM approach.

Previous studies indicated that GP is superior in predicting polygenic traits compared with marker-assisted selection (MAS) based on QTL mapping (Arruda et al. 2016; Alemu 2024) and could reduce selection cycles and enhance genetic gain, aligning with the objectives of plant breeders. We found the similar findings in our research, and our results could be path forward for our next study of other complex traits of watermelon.

## Conclusions

This study highlights the variability in prediction accuracy across populations, influenced by heritability, population type and statistical models. The results in this study suggest that GP could be useful in breeding programs aiming to incorporate the polygenic trait of *Fon* race 2 resistance into watermelon cultivars using the GBLUP and RF models which returned the highest PA values. Our GP from the two populations showed a moderate to high level of accuracy surpassed the QTL mapping explanation of the trait variance. The present study underscores the significance of employing GP in breeding programs aimed to incorporate *Fon* race 2 resistance into watermelon cultivars, particularly when trait correlations are positive. In the present study, we used the GP and QTL data to identify and select the most superior families with *Fon* race 2 resistance. The families selected here, having the highest GEBVs and all QTL associated with *Fon* race 2 resistance could be a viable source for improving elite watermelon cultivars.

## Data Availability

The datasets generated and/or analyzed in this study are available from the corresponding author upon reasonable request.
